# Cabozantinib-Loaded PLGA Nanoparticles: A Potential Adjuvant Strategy for Surgically Resected High-Risk Non-Metastatic Renal Cell Carcinoma

**DOI:** 10.3390/ijms232012634

**Published:** 2022-10-20

**Authors:** Hye Won Lee, Hee Seung Seo, Seon-Yong Yeom, Se-Na Kim, Cho Rim Kim, Dae-Hwan Park, Wooram Park, Young Bin Choy, Chun Gwon Park, Seong Il Seo

**Affiliations:** 1 Department of Urology, Center for Urologic Cancer, National Cancer Center, Goyang 10408, Korea; 2 Department of Biomedical Engineering, SKKU Institute for Convergence, Sungkyunkwan University, Suwon 16419, Korea; 3 Department of Intelligent Precision Healthcare Convergence, Sungkyunkwan University, Suwon 16419, Korea; 4 Department of Urology, Samsung Medical Center, Sungkyunkwan University School of Medicine, Seoul 06351, Korea; 5 Institute of Medical & Biological Engineering, Medical Research Center, Seoul National University, Seoul 03080, Korea; 6 Interdisciplinary Program in Bioengineering, College of Engineering, Seoul National University, Seoul 08826, Korea; 7 Department of Engineering Chemistry, College of Engineering, Chungbuk National University, Cheongju 28644, Korea; 8 Department of Industrial Cosmetic Science and Department of Synchrotron Radiation Science and Technology, College of Bio-Health University System, Chungbuk National University, Cheongju 28644, Korea; 9 Department of Integrative Biotechnology, College of Biotechnology and Bioengineering, Sungkyunkwan University, Suwon 16419, Korea; 10 Department of Biomedical Engineering, Seoul National University College of Medicine, Seoul 03080, Korea; 11 Biomedical Institute for Convergence at SKKU (BICS), Sungkyunkwan University, Suwon 16419, Korea

**Keywords:** adjuvant, cabozantinib, drug delivery, enhanced permeability retention, nanoparticle, poly(lactic-co-glycolic acid), post-surgery metastasis, renal cell carcinoma

## Abstract

Patients with high-risk non-metastatic renal cell carcinoma (RCC) are at risk of metastatic relapse following nephrectomy. Cabozantinib (CZ), a potent multitarget tyrosine kinase inhibitor, interferes with angiogenesis and immunosuppression associated with surgery-induced metastasis. Here, we explored the therapeutic potential of CZ-loaded poly(lactic-co-glycolic acid) (PLGA) nanoparticles (CZ-PLGA-NPs) as an adjuvant strategy for targeting post-nephrectomy metastasis. A clinically relevant subline recapitulating post-nephrectomy lung metastasis of high-risk human RCC, namely Renca-SRLu5-Luc, was established through in vivo serial selection of luciferase-expressing murine RCC Renca-Luc cells. CZ was encapsulated into PLGA-NPs via the conventional single emulsion technique. The multifaceted preclinical antimetastatic efficacy of CZ-PLGA-NPs was assessed in Renca-SRLu5-Luc cells. CZ-PLGA-NPs with a smooth surface displayed desirable physicochemical properties, good CZ encapsulation efficiency, as well as controlled and sustained CZ release. CZ-PLGA-NPs exhibited remarkable dose-dependent toxicity against Renca-SRLu5-Luc cells by inducing G2/M cell cycle arrest and apoptosis. CZ-PLGA-NPs attenuated in vitro colony formation, migration, and invasion by abrogating AKT and ERK1/2 activation. An intravenous injection of CZ-PLGA-NPs markedly reduced lung metastatic burden and prolonged lifespan with favorable safety in the Renca-SRLu5-Luc experimental lung metastasis model. The novel CZ-PLGA-NPs system with multifaceted antimetastatic effects and alleviating off-target toxicity potential is a promising adjunctive agent for patients with surgically resected high-risk RCC.

## 1. Introduction

Surgical tumor resection is the standard treatment for high-risk localized and locally advanced renal cell carcinoma (RCC) [1,2,3,4]. However, patients with this condition are at significant risk of metastatic relapse following nephrectomy (40–60%), which is also reflected by the low five-year metastasis-free and overall survival rates of 31.2% and 44.0%, respectively [4,5,6,7,8,9,10]. Therefore, effective adjuvant therapies that can ultimately improve survival outcomes are warranted, particularly for RCC patients who are at high risk of distant metastasis. Molecular targeted therapies for metastatic RCC, such as vascular endothelial growth factor (VEGF)–targeting tyrosine kinase inhibitors (TKIs)—and inhibitors of the mechanistic target of rapamycin have been extensively tested as adjuvant therapies aiming to eliminate micro-metastasis that may be present at the time of the nephrectomy [6,7,11,12,13,14,15,16,17,18,19,20,21]. Unfortunately, in the adjuvant setting, multiple meta-analyses failed to identify such TKIs that can demonstrate an overall survival benefit in surgically resected RCC with a high risk of metastatic relapse [5,6,7,8,9,10,18,19,20,21,22,23,24,25]. Given the high risk of high-grade adverse events and the higher frequency of treatment withdrawal leading to suboptimal dose-intensity of adjuvant TKIs [7,17,19,20,24], there is currently no globally accepted standard adjuvant TKIs for non-metastatic high-risk RCC patients following nephrectomy [5,6,7,8,9,10,12,13,14,15,16,18,19,20,21,22,23,24,25]. Furthermore, long-term exposure to oral TKIs is associated with decreased drug concentration in the plasma and tumor tissues over time, thereby causing a reduction in the efficacy and therapeutic failures of TKIs [26].

Despite the curative potential of surgical excision of the primary tumor, surgery-induced stress responses are associated with metastasis-promoting effects due to tissue disruption and leakage of residual viable cancer cells into the lymphovascular system, the release of neuroendocrine and stress hormones, tissue hypoxia, extracellular matrix remodeling, neovascularization, and immunosuppression following surgical injury [11,27,28,29,30,31,32,33]. In addition to the angiogenic switch, a reduced number of circulating dendritic cells, a significant dysfunction of cytotoxic T and natural killer cells, an increased release of anti-inflammatory cytokines, and an expansion of immunosuppressive myeloid-derived suppressor and regulatory T cells, which are induced in response to surgical stress, are known as key mechanisms responsible for the reactivation of pre-existing dormant micrometastases and the metastatic colonization of disseminated cancer cells [11,27,28,29,30,31,32,33]. Taken together, an integrated approach combining anti-angiogenesis and immune modulation that can boost the anti-tumor activity is an optimal adjuvant strategy for surgically resected high-risk RCC.

RCC management has advanced significantly with the advent of newer generation TKIs, such as cabozantinib (CZ), that are effective in the first-line setting for intermediate- or poor-risk metastatic RCC, with significant clinical benefits in the progression-free survival and overall response rate [34,35,36,37,38,39,40,41,42,43,44]. CZ is an oral, potent multi-target TKI that targets the VEGF receptor (VEGFR)-2 and several other receptor tyrosine kinases (RTKs), including the hepatocyte growth factor receptor (MET), the TAM (TYRO-3, AXL, and MER) family of receptor kinases, the ROS proto-oncogene 1, the c-kit proto-oncogene product, the Fms-related receptor tyrosine kinase 3, the tropomyosin receptor kinase B, the angiopoietin-1 receptor, and the RET proto-oncogene [34,35,39,41,42,44]. Compared to other pure anti-angiogenic TKIs, the concomitant inhibition of multiple clinically relevant RTKs with a greater potency provided by CZ interferes with tumor progression, metastasis, angiogenesis, and therapeutic resistance to VEGF inhibition through dual multi-facet effects on the tumor cells and their microenvironment [34,35,39,41,42,44]. In particular, CZ has a unique anti-tumor immunomodulatory profile because several targets of CZ, including VEGFR2, MET, and the TAM kinases, play crucial roles in mediating the immunosuppressive tumor microenvironment in metastatic RCC. These multitasking proprieties make CZ a promising adjuvant agent for surgically resected high-risk RCC.

Generally, the recommended starting dose of tablets (Cabometyx) for patients with metastatic RCC is 60 mg once daily, and treatment is continued until the patient is no longer clinically benefiting from therapy or until unacceptable toxicity occurs [26,34,35,38,40,44,45,46,47]. However, despite its successful anticancer efficacy in clinical trials and real-world clinical settings, CZ is associated with a clinically relevant toxicity profile and a high frequency of dose reduction, interruption, or treatment withdrawal leading to sub-optimal dose-induced therapeutic failures [26,34,35,38,40,44,45,46,47]. Furthermore, CZ exhibits poor aqueous solubility and low oral bioavailability, leading to insufficient CZ delivery to tumors below the threshold concentration [26,44,48]. More importantly, chronic long-term exposure to several oral TKIs has been observed to be associated with a decrease in the plasmatic drug concentration over time and, therefore, a reduced efficacy and therapeutic failures of TKIs (‘tachyphylaxis’).

To deal with the main hurdle in the practical application of multi-TKIs, including severe dose-related adverse events owing to particular effects on normal organs, hydrophobicity, and low oral bioavailability in addition to drug resistance, nanoparticles (NPs) based on multi-TKIs have attracted ever-increasing attention in recent years [48]. The enhanced permeability retention (EPR) effect allows for the passive targeting by NPs (ranging from 40 to 400 nm in size) when administrated intravenously due to vascular leakage and defective lymphatic drainage in solid tumors, enabling long-term circulation with increased efficacy and reduced toxicity by decreasing drug accumulation in normal organs [48,49,50,51,52,53]. More importantly, NPs can significantly improve the aqueous solubility and, eventually, the oral bioavailability of poorly soluble drugs due to a smaller size, greater surface area to volume ratio, and higher cell penetration ability [48,54,55]. Currently, several efforts to design systematic formulations to overcome the poor solubility of hydrophobic TKIs have demonstrated that different polymeric nanocomplex NPs significantly improve the anti-cancer efficacy of TKIs, such as sorafenib, ponatinib, and nilotinib, by enhancing their bioavailability and allowing for the efficient delivery of the drug to tumor tissues while reducing adverse side effects [48,49,50,51,52,53,56,57,58,59,60,61].

We therefore hypothesized that translating the multi-TKIs CZ into NP formulations is an ideal strategy as it may increase drug accumulation in tumor cells via the EPR effect and reduce side effects. Poly(lactic-co-glycolic acid) (PLGA), which is a polymer currently approved for clinical usage, hydrolyzes into its monomers (lactic and glycolic acid) and then metabolizes in vivo to be easily excreted from the body [48,49,50,51,52,58,59,60,61]. Therefore, it has been safely used to prepare intravenously injectable NP formulations [48,49,50,51,52,58,59,60,61]. In addition to their biodegradable properties, PLGA synthetic NPs are the most extensively studied of all commercially available polymeric NPs for the delivery of anti-cancer drugs due to their non-immunogenicity, microcytotoxicity, biocompatibility, high drug loading capacity, and controlled slow-release profiles [48,49,50,51,52,58,59,60,61]. This study explored the feasibility and the anti-metastatic efficacy of CZ-loaded PLGA NPs (CZ-PLGA-NPs) as the clinical adjuvant routine following a nephrectomy for high-risk non-metastatic RCC for preventing or reducing post-nephrectomy metastatic relapse. 

## 2. Results and Discussion

### 2.1. Establishment of Post-Nephrectomy Murine RCC Cell Sublines with High Metastatic Potential to the Lung

The lungs are the most common metastatic site in patients with high-risk RCC following a nephrectomy (accounting for up to 60% of metastases), and pulmonary metastatic relapse is a major cause of mortality in these patients [1,2,3,4,62,63]. Herein, we used Renca cells, a well-described murine RCC cell line, as the preclinical model to evaluate the immune-modulatory anticancer therapeutic effects of CZ-PLGA-NPs [64,65,66,67]. The robust myeloid cell infiltrates observed in the in vivo Renca model reflect the immunosuppressive tumor microenvironment associated with more aggressive human RCC phenotypes [64]. Furthermore, the Renca cells spontaneously metastasize to different organs but predominantly to the lungs in a pattern analogous to human RCC, mimicking RCC lung colonization [64,65,66,67]. However, an intravenous injection of parental Renca cells via the tail vein may not accurately reflect human metastatic RCC as it does not precisely mimic several key steps related to metastatic relapse after a nephrectomy in high-risk RCC.

**Figure 1:**
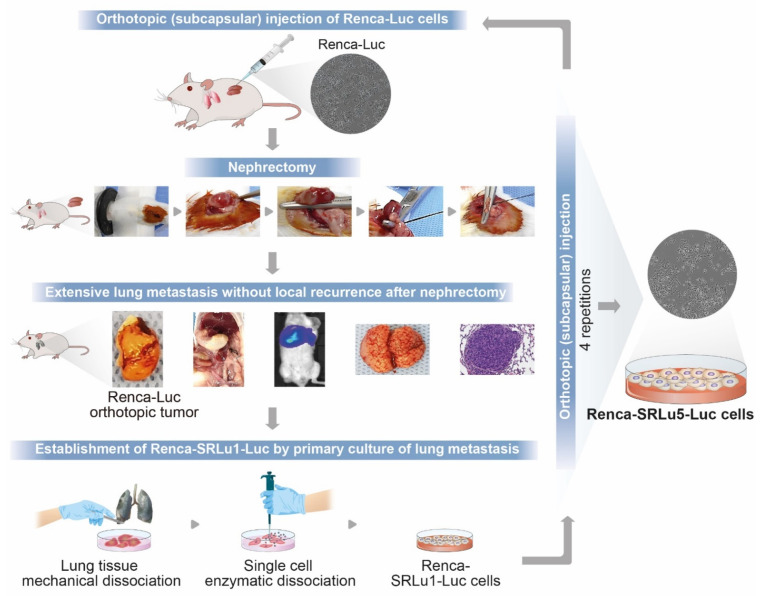
Establishment of the highly pulmonary metastatic murine RCC subpopulation termed Renca-SRLu5-Luc via in vivo serial selection by orthotopic injection and nephrectomy. Schematic representation of the in vivo selection procedures, including subcapsular implantation using a superficial technique, surgical nephrectomy, monitoring of orthotopic tumors, and post-nephrectomy spontaneous lung metastases using bioluminescent imaging, isolation of tumor cells from pulmonary metastases for expansion culture, and reinjection into another mouse.

In order to establish a preclinical model that could recapitulate important hallmarks of the post-nephrectomy pulmonary metastasis from advanced RCC for the development of more effective adjuvant strategies with superior toxicity profiles, an in vivo serial selection was applied to generate a model in which reliable, spontaneous metastatic spread of the tumor to the lungs occurred in a predictable time frame after the orthotopic injection of luciferase-expressing murine RCC Renca-Luc cells and subsequent nephrectomy (Figure 1).

First, RCC orthotopic tumor models were established in BALB/c mice as previously described with some modifications [66,68,69]. Briefly, Renca-Luc cells (5 × 10^5^ cells in 30–40 μL Hank’s balanced salt solution; Gibco, Waltham, MA, USA) were injected into the subcapsular space of the left kidney while preventing the leakage of cells into the surrounding tissue by the careful removal of the needle and immediate swabbing over the injection site with a sterile cotton-tipped applicator for 1 min. For the surgical removal of the primary RCC tumor (d 11), the connecting fat tissue from the caudal end and the adrenal gland from the cranial end were gently dissected from the affected kidney. While gently grasping the kidney, a double knot was made around the ureter, renal artery, and vein using a 5-0 silk suture, and then a cut was made above the knot to remove the kidney. Bleeding from the tied vessels and the ureter was carefully checked. Assessment before and after the nephrectomy (d 10 and 12 post-cell injections, respectively) with in vivo whole-body bioluminescence imaging confirmed the complete removal of the primary renal tumor and no visible metastases post-surgery. However, overt lung metastases were detected in most mice two weeks after nephrectomy. The animals were monitored daily for signs of distress, bleeding, or limited mobility and tolerated well the surgical procedures. The Renca-SRLu1-Luc cells were isolated from a single-cell suspension obtained from pulmonary metastases, which were then reimplanted subrenally into a second set. This process was repeated five times to select the final pulmonary metastatic Renca-Luc cell subpopulation (hereafter named Renca-SRLu5-Luc cells) (Figure 1). We confirmed that all mice developed metastases within a short period (7 d) after the surgical removal of the primary tumor and after the orthotopic injection of Renca-SRlu5-Luc cells.

### 2.2. Physicochemical Characteristics, CZ Entrapment Efficiency, CZ Release Kinetics and Favorable Biocompatibility of CZ-PLGA-NPs

**Figure 2:**
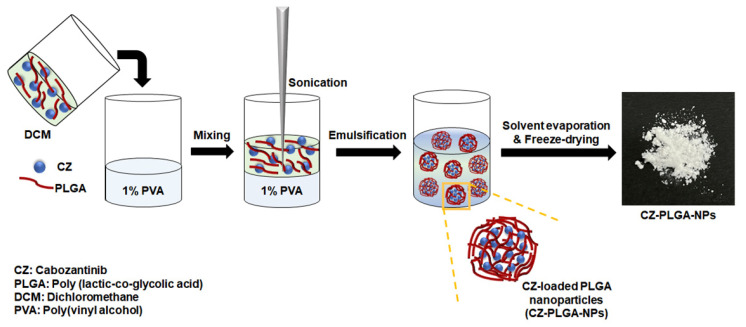
Schematic illustration of the preparation of CZ-PLGA-NPs, including the CZ encapsulation process into PLGA-NPs.

For lipophilic molecules, a simple single emulsification technique is used for encapsulation [70]. CZ-PLGA-NPs were fabricated as reservoir systems (nanocapsule format) composed of a CZ-dissolved liquid core surrounded by a polymeric membrane that controlled the release of the drug using the conventional single emulsification method as previously described (Figure 2) [70]. Briefly, 200 mg PLGA and 30 mg CZ were dissolved in 3 mL dichloromethane and poured into a 10 mL solution of 1% poly(vinyl alcohol). The resulting polymer solution was sonicated for 20 min at 80% amplitude with 1 sec on/off cycles using a Q700 sonicator (Qsonica, Newtown, CT, USA) at 1000 rpm. The prepared emulsion was then maintained at room temperature while stirring for 3 h at a speed of 1000 rpm to evaporate the residual dichloromethane. Next, the CZ-PLGA-NPs were collected via centrifugation and washed with deionized water to remove free CZ. Finally, powdered NPs by freeze-drying (Ilshin Freeze-Dryer FD8508; IlShin Biobase, Dongducheon, Korea) were stored in the deep freezer until further use.

**Figure 3:**
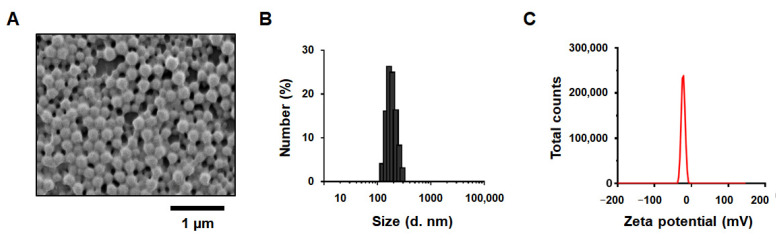
Characterization of physicochemical properties of CZ-PLGA-NPs. (**A**) The surface morphology of CZ-PLGA-NPs with a spherical shape examined using scanning electron microscopy (SEM). Scale bar = 1 µm; (**B**,**C**) Distribution of (**B**) size (diameter) and (**C**) zeta potential values of CZ-PLGA-NPs.

It should be noted that the shape and size of NPs and their surface charges are very important parameters that affect the destiny of in vivo and in vitro behaviors of NPs in physiological conditions [71]. Changes in these properties can have remarkable biological effects on cellular uptake, drug loading and trapping efficiency, as well as the biodistribution and pharmacokinetics of cargo [71]. Relatively less negatively charged anionic (almost neutral) polymeric NPs with small sizes can be more useful for a wide range of biological aspects [71]. The CZ-PLGA-NPs exhibited a spherical shape and a smooth surface (Figure 3A), with a hydrodynamic diameter (particle size) of approximately 210 ± 40 nm (Figure 3B). CZ-PLGA-NPs displayed a negative surface charge (zeta potential) of approximately −22.7 ± 5.0 mV (Figure 3C), confirming that the optimized nanoparticulate formulation of the CZ-PLGA-NPs presented the desired physiochemical properties. 

**Figure 4:**
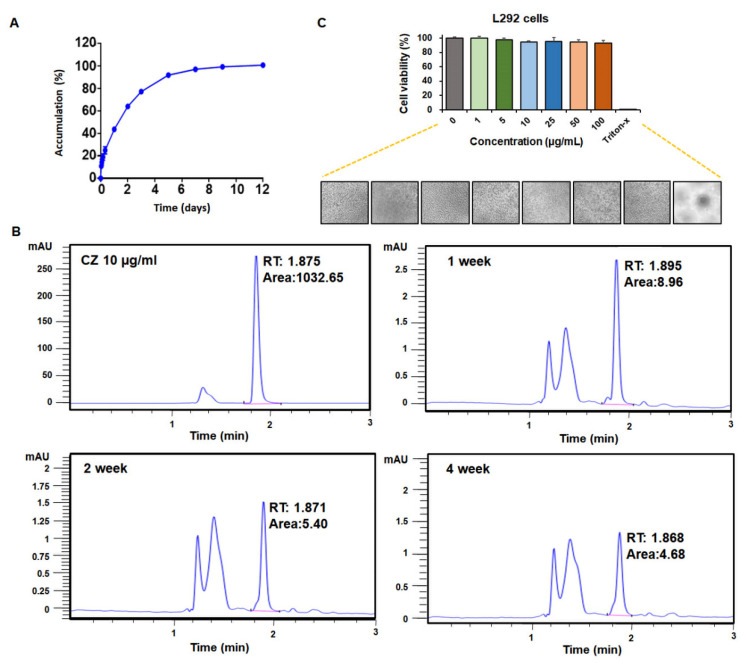
Characterization of CZ release kinetics and cytocompatibility of CZ-PLGA-NPs. (**A**) In vitro CZ release profile of CZ-PLGA-NPs; (**B**) Stability of released CZ in CZ-PLGA-NPs. Representative high-performance liquid chromatogram of released CZ in CZ-PLGA-NPs after one, two, and four weeks. Numerical data represent the mean ± standard deviation (SD) of three independent experiments. (**C**) In vitro cytotoxicity of the drug-free PLGA-NPs on L929 cells and light microscopy images of L929 cells after a 24 h co-incubation.

The PLGA-NPs loaded 57 µg/mg of CZ, with an encapsulation efficiency of 19%, which was released sustainably for up to 12 days (Figure 4A). The chromatograms of CZ-PLGA-NPs at different time points showed well-separated peaks in identical locations as those of the CZ standard solution (Figure 4B), demonstrating that this drug vehicle system retained CZ in a stable form in the PLGA particle matrix, even after 4 weeks of incubation. Finally, the cytotoxic effect on non-tumoral cells is a crucial factor that determines the safety and applicability of new materials. The L929 cell line was selected as a non-tumoral cell line model for the in vitro cytotoxicity evaluation of CZ-PLGA-NPs. Analysis of the in vitro cytocompatibility of drug-free PLGA-NPs showed that PLGA-NPs did not display any cytotoxicity even when the concentration of the PLGA-NPs reached 100 μg/mL (Figure 4C). Taken together, these results indicate that the developed CZ-PLGA-NPs have smooth surfaces and showed desirable particle size, zeta potential, CZ encapsulation efficiency, CZ release kinetics, as well as an appropriate biosecurity profile and high cytocompatibility of the NPs with non-tumoral cells. 

### 2.3. CZ-PLGA-NPs Exhibit High Cytotoxicity toward RCC Cells and Attenuate Their Metastatic Properties

**Figure 5:**
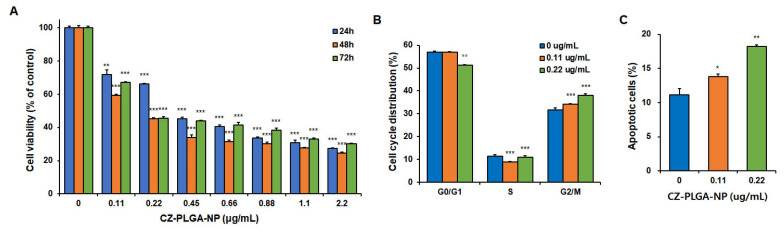
In vitro effects of CZ-PLGA-NPs on the cell viability, cell-cycle progression, and apoptosis of Renca-SRLu5-Luc cells. (**A**) In vitro cytotoxicity assay of CZ-PLGA-NPs against Renca-SRLu5-Luc cells incubated with different concentrations of CZ-PLGA-NPs for 24, 48, and 72 h; (**B**) Effect of CZ-PLGA-NPs on the cell cycle distribution of Renca-SRLu5-Luc cells as determined by flow cytometry based on propidium iodide (PI) staining; (**C**) Effect of CZ-PLGA-NPs on apoptosis of Renca-SRLu5-Luc cells determined using flow cytometry based on the Annexin V–FITC/PI double staining. Each point represents the mean ± SD of three independent experiments. * *p* < 0.05; ** *p* < 0.01; *** *p* < 0.001.

Next, we evaluated the in vitro effects of CZ-PLGA-NPs on multiple biological functions related to the metastasis of RCC. The in vitro treatment of Renca-SRLu5-Luc cells with CZ-PLGA-NPs led to remarkably selective and dose-dependent cytotoxic effects with the drastic inhibition of cell growth (Figure 5A). In particular, CZ-PLGA-NPs treatment for 48 h markedly increased the fraction of cells in the G2/M phase of the cell cycle and decreased cells in the S phase compared to those in controls (Figure 5B and Figure S1A), which was consistent with the partial arrest at the G2/M phase of the cell cycle. Additionally, CZ-PLGA-NPs significantly promoted the apoptosis of Renca-SRLu5-Luc cells (Figure 5C and Figure S1B).

### 2.4. CZ-PLGA-NPs Exhibit Antimetastatic Potential against Metastatic RCC Cells and Activates AKT and ERK in Renca-SRLu5-Luc Cells

**Figure 6:**
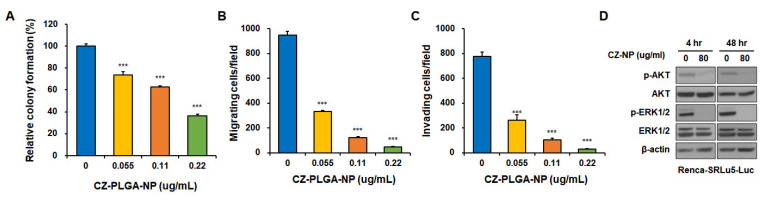
Effects of CZ-PLGA-NPs on in vitro metastatic traits and downstream activation of AKT and ERK in Renca-SRLu5-Luc cells. (**A**) Statistical analysis of the cell colony formation rate in CZ-PLGA-NPs-treated Renca-SRLu5-Luc cells; (**B**,**C**) Statistical analysis of the results from the migration assay (**B**) and the invasion assay (**C**) in CZ-PLGA-NPs-treated Renca-SRLu5-Luc cells; (**D**) Effects of CZ-PLGA-NPs on ERK and AKT signaling molecules in Renca-SRLu5-Luc cells. Renca-SRLu5-Luc cell lysates were isolated at 10 min or 24 h after exposure to indicated concentrations of CZ-PLGA-NPs, and then the protein levels of phosphorylated and dephosphorylated ERK and AKT were analyzed using Western blotting. β-actin was used as a loading control. Each point represents the mean ± SD of three independent experiments. *** *p* < 0.001.

In vitro treatment with CZ-PLGA-NPs significantly diminished the capacity of Renca-SRLu5-Luc cells to form colonies (Figure 6A), migrate (Figure 6B and Figure S2A), and invade (Figure 6C and Figure S2B), suggesting that CZ-PLGA-NPs hold antimetastatic potential against metastatic RCC cells in vitro. Furthermore, we evaluated the effects of CZ-PLGA-NPs on the critical mitogen-activated protein kinase and phosphatidylinositol-3-kinase-protein kinase B (AKT) signaling pathways, which are potential downstream oncogenic effectors targeted by CZ. Consistent with previous data that reported decreased AKT and extracellular signal-regulated kinase (ERK) phosphorylation in Renca cells induced by CZ [41], treatment with CZ-PLGA-NPs effectively abrogated the activation of AKT and ERK1/2 in Renca-SRLu5-Luc cells after 4 h and 48 h of treatment (Figure 6D). These findings demonstrate that CZ-PLGA-NPs exert prominent anticancer effects by preventing key multiple RTK-downstream molecular signals involved in cell proliferation, survival, cancer stemness, and metastatic traits, consistent with the previously reported preclinical anti-tumor activity of CZ [41].

### 2.5. CZ-PLGA-NPs Effectively Attenuate the Lung Metastatic Ability of RCC In Vivo Associated with Prolonged Survival Span

Nanocarriers have traditionally been designed to be selectively entrapped and accumulated around tumor cells and tumor microenvironments within tumor tissues via the EPR effect or release drugs safely and specifically to those cells to increase the bioavailability of the drug while minimizing off-target effects on normal tissues [48,49,50,51,52,53,56,58]. Loading CZ into PLGA-NPs may improve the solubility of poorly water-soluble CZ during systemic delivery and is useful for sustained and controlled CZ release over days, weeks, or months as a stable form, leading to a longer circulation time compared to that of conventional devices. However, high interstitial fluid pressure, hypoxia, acidosis, dense extracellular matrix, hyperpermeability, and occluded or embolized blood vessels associated with pathologic angiogenesis in solid tumors are major obstacles to NP delivery in tumor tissues [48,49,50,51,52,56,57,58,72]. Anti-angiogenic CZ-PLGA-NPs may also decrease the blood flow stasis, allowing more NPs to penetrate the blood vessels and reach the interstitial tissue, thus enhancing their therapeutic effects. 

As previously mentioned, we employed Renca-SRLu5-Luc sublines that successfully demonstrated highly efficient, reliable, and enhanced lung metastasis within a predictable time frame after orthotopic injection and subsequent nephrectomy, mimicking post-nephrectomy lung metastases in high-risk RCC. To focus on post-nephrectomy RCC-associated pulmonary metastasis and to exclude significant morbidity by primary tumors and unpredictable metastases to multiple sites, leading to a specific challenge to quantitative assessment of in vivo anti-metastatic efficacy, a classic experimental lung metastasis assay based on tail vein injection of Renca-SRLu5-Luc cells was performed. 

**Figure 7:**
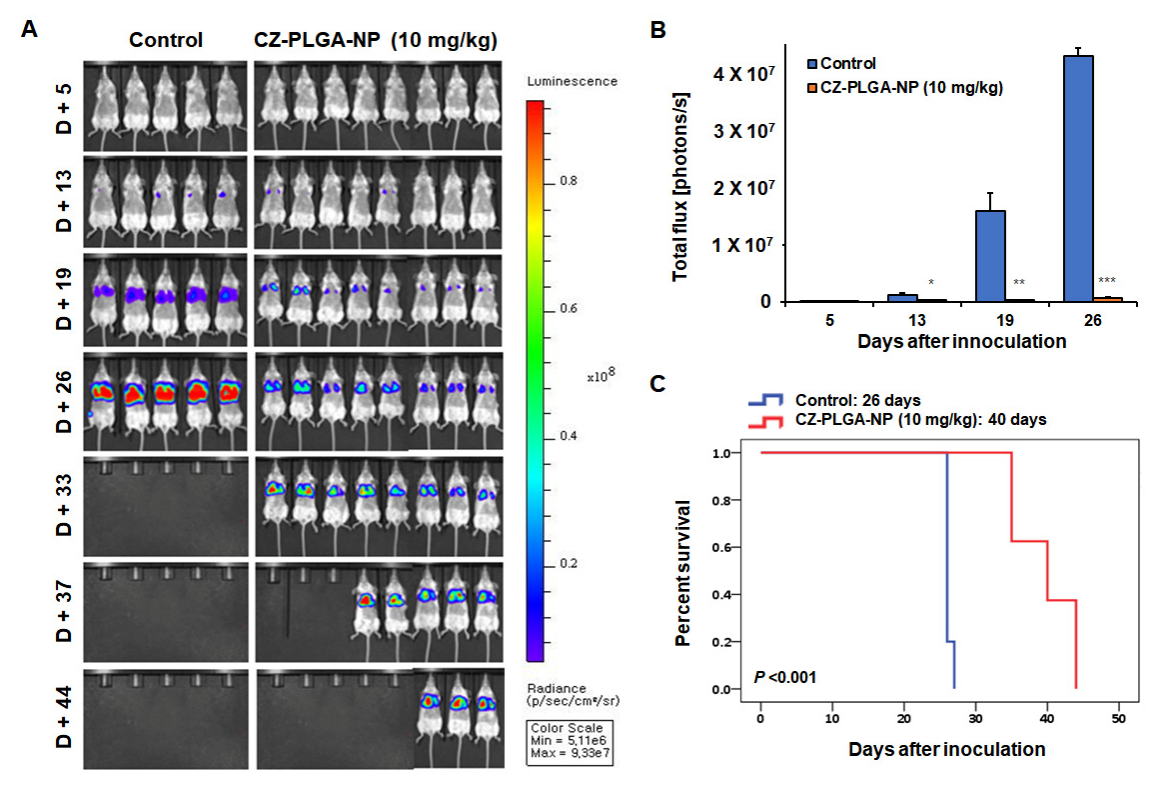
In vivo anti-tumor activity of CZ-PLGA-NPs against Renca-SRLu5-Luc RCC lung metastasis. (**A**) Longitudinal images of the quantification of the metastatic lung tumor growth (lung metastatic burden) based on the whole-body bioluminescence signal, measured as photons/sec. **(B)** The photon flux levels in the different groups of mice were measured using the in vivo imaging system (IVIS). All groups, *n* ≥ 5; data are presented as the mean ± standard error of the mean; * *p* < 0.05; ** *p* < 0.01; *** *p* < 0.001; treated vs. control groups. (**C**) Kaplan–Meier survival curve and comparison of median survival between control (no treatment, n = 5) and treatment groups (in vivo administration of CZ-PLGA-NPs 10mg/kg, twice a week, a total of eight injections during the study, n = 8) (log-rank test).

**Figure 8:**
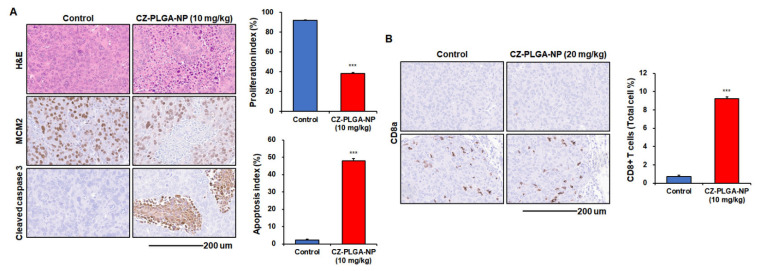
In vivo effects of CZ-PLGA-NPs on Renca-SRLu5-Luc tumor cell proliferation, apoptosis, and CD8+ T cell infiltration into lung metastasis in an experimental Renca-SRLu5-Luc lung metastasis model. (**A**) Representative images of hematoxylin–eosin (H&E) and immunohistochemical (IHC) staining for MCM2 and cleaved caspase-3 in Renca-SRLu5-Luc lung metastases (right panel). Proliferative cells stained with MCM2 and apoptotic cells stained with anti-cleaved caspase-3 antibodies in tumors. Positive staining appears as a brown color. Scale bar = 100 µm. Quantification of IHC staining for MCM2 and cleaved caspase-3 (left panel); (**B**) Representative IHC staining (anti-CD8α+, brown color, right panel) and quantitative analysis (left panel) of cytotoxic CD8+ T cell infiltrates into the lung metastases in the Renca-SRLu5-Luc model. Scale bar = 100 µm. All groups, *n* ≥ 5; data are presented as the mean ± standard error of the mean. *** *p* < 0.001; treated vs. control groups. Scale bars = 200 mm.

In current clinical practice, the CZ tablet formulation (Cabometyx™) was approved at a dose of 60 mg daily for treating advanced RCC. CZ at 2–2.5 mg/kg per day is equivalent to 15–20 mg per day in humans [67,73], and previous preclinical in vivo studies using oral CZ monotherapy (once daily oral gavage of 10–60 mg/kg CZ) have demonstrated its anticancer effects in mouse models of various tumor types, including RCC Renca cell lines [41,67]. For example, a recent study using the subcutaneous Renca model demonstrated the CZ (10 mg/kg, once per day by gavage for 14 days)-mediated tumor growth inhibition through triggered infiltration of both antineoplastic neutrophils/T cells into the tumor bed and significantly enhanced the cytotoxicity of tumor-infiltrated T cells [67]. As therapeutic NPs have generally been injected intravenously every 3–4 days in previous preclinical studies [41], we chose the intravenous treatment of CZ-PLGA-NPs (10 mg/kg, twice a week) in our preliminary study, although a further optimization of in vivo dose and IV injection schedule is required in future studies. Overall, the intravenous treatment with eight cycles of CZ-PLGA-NPs markedly reduced the lung metastatic burden induced by Renca-SRLu5-Luc cells (Figure 7A,B) and significantly improved the overall survival compared to that in the controls (median survival time, 40 vs. 26 days, respectively; *p* < 0.001 by log-rank test) (Figure 7C). CZ-PLGA-NPs treatment was well tolerated in vivo with no toxicity, as evidenced by stable body weights during the treatment period (Figure S3). Further analyses of the Renca-SRLu5-Luc-induced tumors revealed that CZ-PLGA-NPs treatment significantly reduced cancer cell proliferation and increased cancer cell apoptosis, as represented by the lower percentage of the minichromosome maintenance complex component 2 (MCM2) positive cells and the increased fraction of cells with higher levels of cleaved caspase-3 compared to that in the controls (Figure 8A). 

Various targets of CZ are associated with immunosuppression, thus allowing it to directly immunomodulate the tumor microenvironment, increase cytotoxic T cell activation/infiltration, and induce susceptibility of tumor cells to cytotoxic T cell-mediated tumor cell killing, differentiating itself from other VEGF-targeting TKIs [26,37,39,41,43,44,67,74]. In particular, TAM kinases such as AXL and MET promote immunosuppressive phenotypes in tumor-associated immunosuppressive cells (including regulatory T cells, myeloid-derived suppressor cells, and tumor-activated macrophages) [37], which in turn contribute to the resistance against immune checkpoint inhibitors [37,41]. The combined AXL, VEGFR, and MET inhibition appears to be a relatively unique characteristic of CZ; hence, it is unsurprising that CZ monotherapy promotes unique immunomodulatory effects on innate and adaptive anticancer immunity in advanced RCC [26,37,39,41,43,44,67,74]. Interestingly, CZ-PLGA-NPs treatment significantly promoted the infiltration of cytotoxic CD8^+^ T cells into the metastatic lung bed (Figure 8B), suggesting that CZ-PLGA-NPs may mechanistically support the infiltration of cytotoxic CD8^+^ T into the tumor microenvironment, enhancing anti-tumor T cell immunity and contributing to the observed reduction in RCC lung metastasis growth. These results are consistent with previous findings suggesting that the in vivo immune-modulating effects of CZ on Renca cells are related to the activation of both neutrophil-mediated innate immunity and T cell-mediated adaptive immunity [37,39,41,43,44,67,74].

Despite the extensive efforts to identify effective adjuvant therapies, no drugs are recommended for routine clinical use in high-risk RCC due to their poor benefit-to-harm ratios [5,8,9,12,13,14,15,16,18,19,22,23,24,25]. Further research on the nano-delivery of antimetastatic TKIs for toxicity reduction and efficacy enhancement may be increasingly important. To our knowledge, no studies have reported CZ-loaded NPs to overcome the limitations of CZ, including poor solubility, low oral bioavailability, and high toxicity burden associated with a challenge for a clinical translation of CZ into an antimetastatic adjuvant TKI in high-risk RCC. In the present study, we focused on PLGA among diverse polysynthetic materials because PLGA has been approved by the US Food and Drug Administration and the European Medicines Agency for pharmaceutical applications via parenteral routes, suggesting that PLGA-based NPs are in a better position for clinical trials compared to that of other NP systems [48,55]. Loading CZ into PLGA-NPs provides many additional advantages, such as passive targeting via the EPR effect, increased specific surface area, high backbone stability, controllable slow release according to degradation kinetics, micro-cytotoxicity, high drug loading, and feasibility of modification of morphology/structure to increase tumor targeting [48,49,50,51,52,58,59,60,61]. Moreover, PLGA NPs are highly appreciated for their preeminent immunomodulation to enhance the anti-tumor immune response through the continuous stimulation of immune cells, resulting in a rapid increase in the infiltration of CD8+ T cells, reducing regulatory T cells and the establishment of anti-tumor immune memory. Finally, loading anti-angiogenic TKIs such as CZ into PLGA-NPs can increase the solubility of poorly water-soluble CZ during systemic delivery and, to some extent, decrease the blood flow stasis, allowing for more NPs to penetrate the blood vessels and reach the interstitial tissue, thereby exacerbating their therapeutic effects.

**Figure 9:**
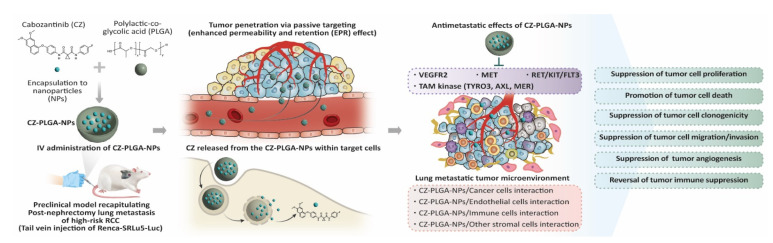
Graphical representation of potential models of anti-tumor activity of CZ-PLGA-NPs against Renca-SRLu5-Luc RCC lung metastasis.

The current study has illustrated the use of PLGA nanocarriers to improve the in vitro and in vivo antimetastatic activity of CZ, and it may therefore be a potential adjunctive antimetastatic agent for patients with high-risk RCC following surgical resection. This study demonstrates a predominant effect of CZ-PLGA as an antimetastatic agent in vivo, suggesting the potential of employing nanocarriers to improve the delivery and efficacy of small molecular antimetastatic drugs. The excellent in vivo antimetastatic effects and biocompatibility of CZ-PLGA-NPs in the present study (Figure 9) are consistent with several reports on the anti-proliferative, pro-apoptotic, and immune-modulating effects of CZ in Renca in vivo models [41,67,74]. The unique size, EPR effects, and tumor extravasation of CZ-PLGA-NPs promote them as attractive carriers for passive drug targeting. Our preclinical experiments demonstrate that incorporating CZ into the PLGA-NPs system increases the delivery efficiency of CZ to RCC lung metastatic cells with minimal effects on non-cancerous cells. CZ-PLGA-NPs significantly prevented Renca-SRLu5-Luc cells from settling in the lung niche, thereby delaying the development of lung metastases and prolonging the lifespan of the animals. We believe our study significantly contributes to the literature because we provide in vivo and in vitro support, using novel clinically relevant models, for PLGA-NPs as a valuable CZ delivery system, which is a promising adjuvant strategy for suppressing post-nephrectomy metastatic relapse in patients with high-risk RCC. 

For improving the success rate of CZ-PLGA-NPs’ clinical translation from preclinical models, it would be beneficial if in-depth investigations to strengthen our findings were performed through further studies. A follow-up study should comprehensively compare the multifaceted preclinical effects of CZ-PLGA-NPs between free CZ, unloaded PLGA-NPs, and CZ-PLGA-NPs. Cellular uptake kinetics, the biodistribution, pharmacokinetic/pharmacodynamic characteristics of IV-administered CZ-PLGA-NPs, and the optimization of treatment regimen (dose/injection schedule) remain to be thoroughly evaluated for better insights into its unique efficacy and safety profiles through affecting both tumor cells and tumor microenvironments. The further optimization of CZ-based adjuvant treatment for patients with high-risk RCC requires identifying additional biomarkers related to the response, resistance, treatment-related toxicity, and exploration of complementary drug targets. Consequently, the integration of risk stratification based on genomic and molecular biomarkers is urgently needed in future RCC adjuvant clinical trials using CZ to improve the identification of patients at very high risk of metastatic relapse, designing a more tailored approach, and potentially yielding greater benefits from adjuvant CZ. Moreover, adjuvant CZ may be re-envisioned using immunohistochemical and genomic tools to investigate novel strategies that discriminate between immune-mediated and VEGF-dependent tumors, providing a more personalized patient treatment. 

## 3. Materials and Methods

### 3.1. Formulation, Development, and Characterization of CZ-PLGA-NPCZ-PLGA-NPs

CZ (XL184, BMS-907351) was purchased from Selleck Chemicals (Houston, TX, USA). Dichloromethane, acetonitrile, and dimethylformamide were obtained from Daejung (Seoul, Korea). PLGA (lactic acid:glycolic acid, 1:1; viscosity, 0.55–0.75 dL/g) was obtained from Durect Corporation (Cupertino, CA, USA). Poly(vinyl alcohol) (87–89% hydrolyzed; molecular weight, 31,000–50,000) and Tween 80 were obtained from Sigma-Aldrich (St. Louis, MO, USA).

Optimized CZ-PLGA-NPs were diluted in deionized water for morphological characterizations. To prepare samples for scanning electron microscopy (JEM-7600F; JEOL, Akishima, Tokyo, Japan), a drop of the diluted solution was placed on a copper grid and then dried in a hum hood. Next, the grid was placed on a sample holder to evaluate the NPs’ surface morphology. The average particle size and zeta potential of CZ-PLGA-NPs were determined using dynamic light scattering with a Zetasizer Nano ZS90 (Malvern Panalytical, Malvern, UK). A 12 mm square polystyrene disposable cuvette (DTS0012; Malvern Panalytical) was used to measure the particle size. Sizes were analyzed using the Zetasizer software by averaging 30 measurements. CZ-PLGA-NPs were equilibrated for 120 s, and the measurements were performed at 25 °C. Folded capillary zeta cells (DTS1070; Malvern Panalytical) were used to measure the zeta potential of CZ-PLGA-NPs using the Zetasizer software (Malvern Panalytical). The results are expressed as the mean of five measurements ± standard deviation.

### 3.2. Determination of Drug Loading and Encapsulation Properties

To measure the loaded amount of CZ, 5 mg of CZ-PLGA-NPs was suspended in 5 mL of dimethylformamide. The concentration of CZ was measured using high-performance liquid chromatography (HPLC; 1260 series HPLC system; Agilent Technologies, Santa Clara, CA, USA) using a C18 column (5 μm, 4.6 × 250 mm; Agilent Technologies) under the following conditions: flow rate, 0.5 mL/min; injection volume, 20 μL; and column temperature, 30 °C. For this experiment, the mobile phase was a 40:60 (*v*/*v*) mixture of 20 mM ammonium acetate (pH 3.2) and acetonitrile, respectively. The encapsulation efficiency (EE) of the CZ-PLGA-NPs was calculated as follows: *EE* (%) = (amount of drug loaded into the NPs / initial amount of CZ) × 100.

### 3.3. CZ Release Profile and Stability

To evaluate the drug release kinetics of CZ-PLGA-NPs, a suspension of 5 mg CZ-PLGA-NPs in 3 mL of phosphate-buffered saline (PBS; pH 7.4) containing 0.1% (*w*/*v*) Tween 80 was placed in a dialysis membrane bag (Snake Skin^TM^ Dialysis Tubing, 10 kDa; Thermo Fisher Scientific, Waltham, MA, USA). The bag was then immersed in 47 mL of PBS (pH 7.4) containing 0.1% (*w*/*v*) Tween 80 to meet the sink condition of CZ and incubated at 37 °C with gentle agitation (160 rpm) in a shaking incubator (Puricell Shaking 80; Cryste Novapro Co., Seoul, Korea). At selected time intervals, 20 mL of the released medium was collected and replaced with fresh medium. The concentration of CZ in the collected medium was measured using HPLC, as described in Section 2.2. The experiment was performed in triplicate. 

To assess the stability of the released CZ, 1 mg/mL CZ-PLGA-NPs was immersed in PBS (pH 7.4) and incubated at 37 °C with gentle agitation (160 rpm) in a shaking incubator (Puricell Shaking 80, Cryste Novapro Co.) for one, two, and four weeks. At selected time intervals, the supernatant was isolated by centrifugation (27,000× *g*, 10 min), and the amount of CZ in each sample was further analyzed by HPLC as previously described. The data are represented as the percentage of cumulative CZ release vs. time. The chromatograms of the CZ-PLGA-NPs were compared with those of a CZ standard solution to determine the stability of the CZ released from the NPs.

### 3.4. Cell Culture

Murine RCC Renca cells were purchased from the American Type Culture Collection (Manassas, VA, USA), expanded, and frozen in liquid nitrogen at a low passage number. To allow for easy tracking of metastasis formation derived from Renca-induced tumors, cells were stably transduced with lentiviral expression particles harboring the bioluminescent molecule firefly luciferase (LVP325; Amsbio, Cambridge, MA, USA) as previously described [75]. Renca, Renca-Luc, and Renca-SrLu5-Luc cells were maintained in RPMI-1640 medium supplemented with 10% fetal bovine serum (FBS) and 1% penicillin-streptomycin (both from Gibco) at 37 °C in a 5% CO_2_ humidified incubator. To prevent cross-contamination, all cell lines were authenticated by short tandem repeat profiling and checked periodically for mycoplasma using the EZ-PCR Mycoplasma Test Kit (GeneAll Biotechnology, Seoul, Korea).

### 3.5. Toxicity of PLGA-NPs

Mouse connective tissue fibroblasts (L929 cells; Korean Cell Line Bank, Seoul, Korea) were cultured in RPMI-1640 medium supplemented with 10% FBS and 1% penicillin-streptomycin (Gibco). For the cytotoxicity assays, L929 cells were seeded onto 96-well plates (1 × 10^3^ cells/well) and incubated for 24 h. Next, the wells were washed thrice with PBS, and fresh medium was added with 100 µL of empty PLGA-NPs prepared in the same culture medium at varying particle concentrations (1, 5, 10, 25, 50, and 100 μg/mL). After 24 h incubation, the wells were washed thrice with PBS, and fresh media was added. Finally, cytotoxicity was measured using the D-Plus CCK cell viability assay kit (Dongin Biotech Co., Seoul, Korea) according to the manufacturer’s instructions. Briefly, cell counting kit-8 (CCK-8) solution was added to the wells and incubated for 2 h at 37 °C. The absorbance of the cells at 450 nm was measured using a microplate reader (Synergy HTX; BioTek Instruments, Winooski, VT, USA).

### 3.6. Analysis of Cell Growth and Viability

Renca-SRLu5-Luc cells were seeded at a density of 1 × 10^4^ cells in 100 μL of culture medium containing 10% FBS in 96-well plates (cat. #167008; Thermo Fisher Scientific) and allowed to adhere overnight. Untreated cells served as a control while experimental cells were treated with serial dilutions of CZ-PLGA-NPs (0.11, 0.22, 0.45, 0.66, 0.88, 1.1, and 2.2 ug/mL) for 24, 48, and 72 h. Cell viability was evaluated using a CCK-8 assay (Dojindo Laboratories, Kumamoto, Japan). Six wells were included for every control and drug concentration in each experiment, and triplicate experiments were performed. To assess in vitro cell growth, an equal volume of 0.4% (*w*/*v*) trypan blue was added to each cell suspension, and the number of viable cells was determined by dye exclusion using a hemocytometer.

### 3.7. Analysis of Cell Cycle Progression and Apoptosis

For cell cycle analysis, Renca-SRLu5-Luc (5 × 10^5^) cells were seeded in six-well plates and incubated for 16 h. After CZ-PLGA-NP treatment for 48 h, the cells were harvested, washed twice with PBS, and fixed with 70% ethanol at 4 °C for 30 min. The cells were then incubated with propidium iodide (BD Biosciences, Franklin Lakes, NJ, USA) according to the manufacturer’s instructions. Subsequently, DNA staining and cell cycle distribution were performed using the FACSVerse Flow Cytometer and FACSuite software (BD Biosciences). Cell apoptosis was assessed after CZ-PLGA-NPs treatment for 48 h using an Alexa Fluor 488 Annexin V/Dead Cell Apoptosis Kit (Thermo Fisher Scientific) according to the manufacturer’s instructions and analyzed using the FACSVerse Flow Cytometer and FACSuite software (BD Biosciences).

### 3.8. Cell Clonogenicity Assay

Renca-SRLu5-Luc cells (0.5 × 10^3^ cells/well) were seeded on six-well plates and maintained in a complete medium with either dimethyl sulfoxide (vehicle) or various concentrations of CZ-PLGA-NPs (0.055, 0.11, or 0.22 μg/mL) for one week. After staining with 0.1% crystal violet, viable colonies in each well were counted. After images of the cell colonies were obtained, the dye was removed using 10% acetic acid, and the respective absorbance at 570 nm was determined using spectrophotometry.

### 3.9. Analysis of Cell Migration and Invasion

Invasion and migration assays were conducted using Transwell chambers (6.5 mm in diameter, 8 μm pore size; Corning Inc., Corning, NY, USA) with or without Matrigel (Corning) according to the manufacturer’s instructions. Briefly, 1 × 10^4^ Renca-SRLu5-Luc cells were seeded on the upper chamber of the Transwell chamber in RPMI-1640 medium without FBS containing different CZ-PLGA-NPs concentrations (0.055, 0.11, and 0.22 μg/mL). RPMI-1640 medium with 10% FBS and conditioned medium were placed in the lower chamber. After a 48 h incubation, the remaining cells in the upper chamber were removed using a cotton swab. Cells on the lower surface of the membrane were fixed with 4% paraformaldehyde fix solution, stained with 0.5% crystal violet for 30 min, and imaged at 200× magnification. The average number of stained cells was calculated based on the analysis of 10 fields.

### 3.10. Analysis of Oncogenic Signaling Pathways

Renca-SRLu5-Luc cells (5 × 10^6^) were lysed in 1 mL radioimmunoprecipitation assay buffer (150 mM NaCl, 50 mM Tris-HCl [pH 7.2], 0.5% NP-40, 1% Triton X-100, and 1% sodium deoxycholate) containing protease and phosphatase inhibitor cocktails (Sigma-Aldrich). Cell lysates were separated on sodium dodecyl sulfate–polyacrylamide gels and transferred onto an Immobilon-P membrane (Millipore, Burlington, MA, USA). Membranes were blocked using 5% skim milk and 0.1% Tween 20 solution for 1 h and then incubated overnight at 4 °C with specific primary antibodies. Antibodies against β-actin (Santa Cruz Biotechnology, Dallas, TX, USA), p-AKT, p-ERK, AKT, and ERK (Cell Signaling Technology, Danvers, MA, USA) were used. Membranes were then incubated with a horseradish peroxidase (HRP)-conjugated secondary antibody (1:2000 dilution; Cell Signaling Technology) for 1 h and developed using an ECL-Plus Kit (Thermo Fisher Scientific).

### 3.11. In Vivo Experimental Lung Metastasis Assay

For in vivo efficacy evaluation using experimental RCC lung metastasis models, Renca-SRLu5-Luc cells (1.5 × 10^5^ cells/100 μL sterile PBS) were intravenously injected into the tail vein of BALB/c female mice (six to eight weeks old) [68,76]. On day five, mice were randomly divided into two groups (*n* ≥ 5 per group) and intravenously injected with CZ-PLGA-NPs (200 μL, 10 mg/kg) twice per week or untreated (as a control) for four weeks in total (D + 5, D + 9, D + 13, D + 16, D + 20, D + 23, D + 27, and D + 30). As no overt lung metastases were detected using bioluminescent imaging in the mice, treatment starting at this time (D + 5) resembled a form of adjuvant therapy. Lung metastatic tumor burden was determined on days 5, 13, 19, 26, 33, 37, and 44 after implantation via analysis of luciferase luminescence. Briefly, mice were intraperitoneally injected with D-luciferin (150 mg/kg in PBS; PerkinElmer, Waltham, MA, USA) and anesthetized using isoflurane [65]. The photons produced by the luciferase activity of the Renca-SRLu5-Luc cells in the lungs were quantitatively determined 10 min following D-luciferin administration using the In Vivo Imaging System 200 equipped with the Living Image Software package v4.7.3 (Xenogen, Alameda, CA, USA). The animals were monitored for the entire duration of the experiments. Viability checks for mortality and morbidity were performed at least once daily, and detailed clinical signs, including body weight, were collected three times per week. Mice were euthanized according to institutional guidelines using survival endpoints, including signs of distress, labored breathing, abdominal distension, or ≥20% weight loss. All animals were humanely euthanized at the end of the experiments. 

### 3.12. Histological and Immunohistochemical Analyses

The lungs of each in vivo treatment group were harvested and fixed in 10% neutral buffered formalin, paraffin-embedded, and then sectioned (4 μm thickness) for further analyses. The sections were stained with hematoxylin-eosin following standard procedures. For immunohistochemical localization of CD8 alpha (a marker of cytotoxic T cells), MCM2 (a marker of cell proliferation), and cleaved caspase-3 (a marker of apoptosis), the sections were deparaffinized in xylene, rehydrated in graded alcohol, and transferred to 0.01 M PBS (pH 7.4). After heat-induced epitope retrieval using pH 6.0 citrate buffer (for MCM2 and cleaved caspase-3 detection) or pH 9.0 EDTA buffer (for CD8α detection) for 3 min at 121 °C, endogenous peroxidase was blocked using 3% hydrogen peroxide in PBS for 10 min at room temperature. After washing with PBS, the tissue sections were treated with a serum-free blocking solution (Dako, Glostrup, Denmark) for 20 min at room temperature. Subsequently, the samples were incubated overnight at 4 °C with the following primary antibodies: anti-MCM2 rabbit polyclonal antibody (1:2000 dilution; ab4461, Abcam, Cambridge, UK), anti-cleaved caspase-3 rabbit polyclonal antibody (1:400 dilution; #9661, Cell Signaling Technology), or anti-CD8α rabbit monoclonal antibody (1:200 dilution; Cell Signaling Technology). After washing with PBS, the sections were incubated for 30 min at room temperature with HRP-labeled polymer-conjugated secondary antibodies against rabbit IgG (Dako). The color reaction was developed using a ready-to-use 3,3′-diaminobenzidine substrate–chromogen solution (Dako) for 5 min and then washed with distilled water. Lastly, all slides were counterstained with Mayer’s hematoxylin. 

The proliferation and apoptosis indices were calculated based on the number of labeled MCM2 or cleaved caspase-3 cells, respectively. The indices are expressed as percentages of at least 500 cells per region. To determine the number of stained cells in each observed tissue component, three respective areas of the tumor core were evaluated at 400× magnification, and mean values were calculated. Quantification of immunohistochemical CD8 staining was performed to evaluate cytotoxic CD8^+^ T cell infiltration into lung metastases using the Image-Pro Plus software (Media Cybernetics, Rockville, MD, USA) based on three representative visual areas in each section of each tumor. Tumor-infiltrated lymphocytes were counted in all fields of the digital slides at 20× magnification using the Aperio ImageScope software (Leica Microsystems, Wetzlar, Germany).

## 4. Conclusions

In this study, a novel nanoplatform was developed using PLGA to deliver CZ. The CZ-PLGA-NPs demonstrated desirable physicochemical characteristics, excellent encapsulation efficiency, and sustained drug release properties. CZ-PLGA-NPs mediated significant preclinical antimetastatic effects on RCC lung metastatic cells both in vitro and in vivo. Hence, PLGA-NPs loaded with CZ may provide a sustained delivery of the drug into the metastatic lung sites via the EPR effect, resulting in enhanced therapeutic effects and minimal off-target toxicity. Given the demonstrated effectiveness of CZ in a clinically relevant RCC model, we believe that CZ-PLGA-NPs are a promising adjuvant strategy for suppressing post-nephrectomy metastatic relapse in patients with surgically resected high-risk RCC.

## Supplementary Materials

The following supporting information can be downloaded at: https://www.mdpi.com/article/10.3390/ijms232012634/s1.
