# Supplementary material for: Cabozantinib-Loaded PLGA Nanoparticles: A Potential Adjuvant Strategy for Surgically Resected High-Risk Non-Metastatic Renal Cell Carcinoma
Source: Int J Mol Sci. 2022 Oct 20;23(20):12634. doi: 10.3390/ijms232012634 (PMC9604013; doi:10.3390/ijms232012634)
Supplement: Supplementary file 1 [file ijms-23-12634-s001.zip › ijms-1949461-supplementary.pdf]

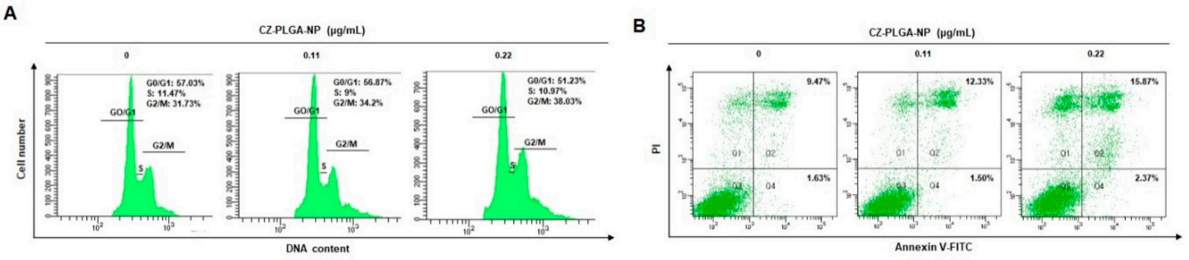

Supplementary Figure S1. In vitro effects of CZ-PLGA-NPs on cell-cycle progression and apoptosis of Renca-SRLu5-Luc cells. (A) Effect of CZ-PLGA-NPs on the cell cycle distribution of Renca-SRLu5-Luc cells as determined by flow cytometry based on propidium iodide (PI) staining. (B) Effect of CZ-PLGA-NPs on apoptosis of Renca-SRLu5-Luc cells determined using flow cytometry based on the Annexin V–FITC/PI double staining.

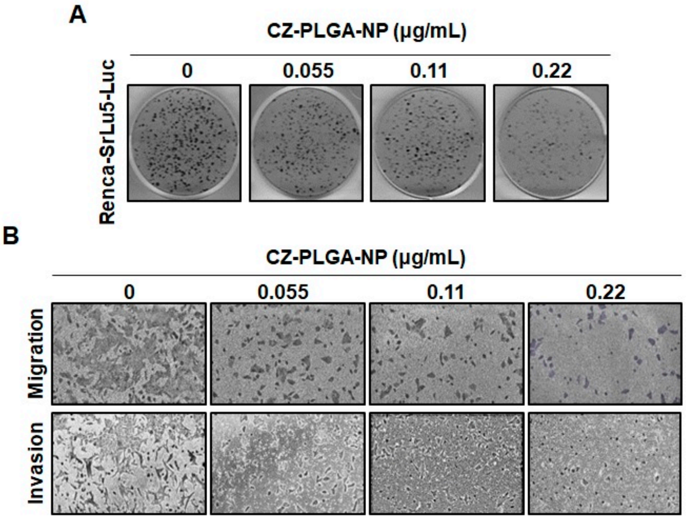

Supplementary Figure S2. In vitro effects of CZ-PLGA-NPs on cell migration and invasion of Renca-SRLu5-Luc cells. Representative images of the results from the migration assay (A) and the invasion assay (B) in CZ-PLGA-NP-treated Renca-SRLu5-Luc cells (scale bar = 200 μm).

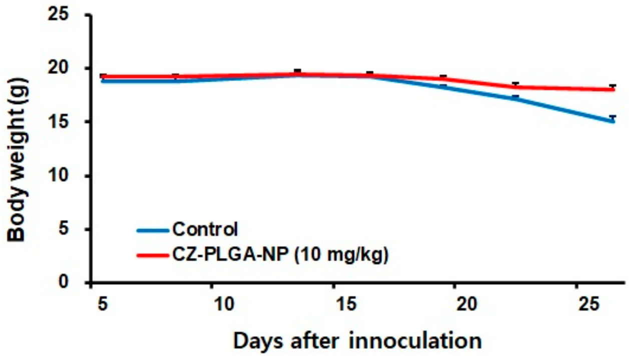

Supplementary Figure S3. In vivo safety of CZ-PLGA-NP in the Renca-SRLu5-Luc RCC lung metastasis model. Body weight of mice in control and treatment groups over four weeks.
